# Bioremediation and pharmacological applications of gold nanoparticles synthesized from plant materials

**DOI:** 10.1016/j.heliyon.2021.e06591

**Published:** 2021-03-29

**Authors:** Sunday Adewale Akintelu, Bo Yao, Aderonke Similoluwa Folorunso

**Affiliations:** a MOE Key Laboratory of Cluster Science, Beijing Key Laboratory of Photoelectronic Electrophotonic Conversion Materials, School of Chemistry and Chemical Engineering, Beijing Institute of Technology, Beijing, 102488, PR China; b Department of Pure and Applied Chemistry, Ladoke Akintola University of Technology, Ogbomoso, Nigeria; c Department of Chemistry, Louisiana State University, Louisiana, USA

**Keywords:** Gold nanoparticles, Synthesis, Plant materials, Bioremediation and pharmacological applications

## Abstract

Nanotechnology and nanoscience are gaining remarkable attention in this era due to their distinctive properties and multi applications. Gold nanoparticles (AuNPs) is one of the most relevant metal nanoparticles with enormous applications in various field of research and industries. The demand for AuNPs is increasing rapidly. Extensive awareness has been allotted to the development of novel approaches for the synthesis of AuNPs with quality morphological properties using biological sources due to the limitations associated with the chemical and physical methods. Several factors such as contact time, temperature, pH of solution media, concentration of gold precursors and volume of plant extract influences the synthesis, characterization and applications of AuNPs. Characterization of synthesized AuNPs is important in evaluating the morphological properties of AuNPs since the morphological properties of AuNPs affect their potential use in various applications. This review highlights various methods of synthesizing AuNPs, parameters influencing the biosynthesis of AuNPs from plant extract, several techniques used for AuNPs characterization and their potential in bioremediation and biomedical applications.

## 1 Introduction

Metal nanoparticles have displayed enormous potential in various applications due to their exceptional catalytic property, anticancer property, medical diagnostic application, antimicrobial activity, biomedical application, sensory application, food preservation, agriculture, pesticide and insecticide application [1, 2, 3]. AuNPs remain dominant and prominent when compared with other metal nanoparticles due to its activities and applications such as; photothermal therapy, drug delivery, immune chromatographic identification, biosensors, photocatalytic and electronics [4,5] Numerous approaches such as physical, chemical and biological methods have been used in the synthesis of AuNPs [6]. The following advantages of biological methods of AuNPs synthesis; (i) been biologically compatible and offer substantial applications in biology and medical field, (ii) It involve the use of natural substances e.g. algae, plants, fungi, microorganisms, (iii) it does not require the use of toxic regents which enhanced its applications in pharmaceutical and biomedical fields, (iv) it is simple to achieve and consume little energy, (v) it is cost effective because no external stabilizing agents are generally required (vii) the possibility of large-scale synthesis is achievable and (viii) reproducibility is production [7, 8, 9, 10] have made this approach more rewarding when compared with other conventional methods. Plants phytochemicals such as carbohydrates, flavonoids, terpenes, alcohol, phenolics, proteins and glycosides have displayed vast potential in reduction of metal ions from their higher oxidation state to low reduction potential [11, 12, 13]. The antioxidant potential of plant's phytochemicals enhances their rapid conversion of gold precursor (chloroauric acid solution) into AuNPs [14,15]. The extensive applications of AuNPs synthesized using plant extracts in drug delivery, tissue imaging and identification of clinical pathogens are due to their antimicrobial activities which are linked with the plant's phytochemicals found in the extracts [16]. Despite the numerous literatures on synthesis, characterization and applications of AuNPs synthesized using plant extracts, a lot of research is still ongoing in this field owing to the diversity and potential of plants in production of AuNPs with different shapes [10,17, 18, 19]. In this review, we provide a concise introduction to the recent development in green synthesis and characterization techniques involved in AuNPs production. We consequently laid emphasis on the recent advances in the bioremediation and biomedical applications of AuNPs biosynthesized from plant materials.

## 2 Methods of synthesizing AuNPs

### 2.1 Physical methods

Evaporation, condensation, high energy ball, milling sputter deposition, pyrolysis, diffusion, laser ablation and plasma arcing are the commonly used techniques associated with the physical methods of synthesizing AuNPs [20]. The use of evaporation-condensation approach for the synthesis of AuNPs involves the use of a tube furnace under atmospheric pressure from which the source material inside a boat centred in the furnace is been vaporized into the carrier gas [21]. Despite the merits of this technique the following are its limitation; large space is needed to accommodate the tube furnace, high amount of energy and time are been wasted in establishing stable thermal condition [22]. The laser ablation is another technique adopted in the physical synthesis of AuNPs, this process occurs in a chamber under vacuum in the presence of inert gases [21]. This approach is advantageous in production of colloidal nanoparticles. Spray pyrolysis, energy ball milling by impact collisions and plasma-arcing in the presence of high temperatures have been used as a physical method for the synthesis of AuNPs [23].

### 2.2 Chemical method

The most recognized techniques used in the chemical methods of synthesizing AuNPs are chemical reduction, thermal decomposition, electrochemical and micro-emulsion techniques. Chemical reduction involve the use of either inorganic or organic reagents as reducing agents. Reports from literatures have shown trisodium citrate dehydrate, elemental hydrogen sodium borohydride, methoxy polyethylene glycol, ascorbate and potassium bitartrate as prominent reducing agents used in chemical reduction technique [22,24]. Micro-emulsion is another form of the chemical method, it occurs in aqueous cores (nanoreactors) of the reverse micelles which are scattered in an organic reagent and stabilized by surfactant [25]. The micro-emulsion techniques is profitable for the production of AuNPs with control homogeneity, morphological and geometrical properties [26]. The electrochemical method uses electricity as the source of electron for the reduction of gold precursor, the electricity also serves as a controlling force during the synthesis. It demands the passage of electric current into two electrodes separated by electrolyte and the synthesized AuNPs occurs at the electrode or at the electrolyte interface [27]. Thermal decomposition has been documented to be the most common chemical techniques, nucleation occurs during this process when the gold precursor (HAuCl_4_ or AuCl_3_) is added into the heated solution in the presence of surfactant and the growth stage happen at a very high reaction temperature [28]. In thermal decomposition process the size of synthesized AuNPs is been determined by reaction temperature, time and surfactant [21]. However, literatures have reported the chemical approaches of AuNPs synthesis using several reducing agents, but some findings revealed that nanoparticles formed from chemical methods show some threats on human health [29].

### 2.3 Biological methods

Many scientist have utilized the potential of biological materials in development of effective, easy to operate, cheap, less toxic, flexible and environmental friendly route for the synthesis of AuNPs. Biological methods which is also referred to as green synthesis, utilized plant extract [30,31], algae [32], mushrooms [33], bacteria (sulphate-reducing bacteria) [34] and truffles [35] in AuNPs synthesis because they contain biochemicals that function as reducing agent to reduce the cytotoxicity associated with the use of expensive and toxic reagents in chemical method of AuNPs synthesis. The chemical constituents of biological materials such as amine, alkaloids, flavonoids, amides, proteins, tannins, carbohydrates are accountable for the reduction of gold precursor because they contain hydroxyl (–OH) functional groups that can donate electrons to the gold ions. The use of microorganisms in the biosynthesis of AuNPs is very beneficial due to the global availability of enzymes, mycelia and fruiting bodies. Nevertheless, this approach is slow, toxic and the high cost of incubation of some organisms are the limitations of this method [36].

The synthesis of AuNPs using mushroom such as *Agaricus bisporus* and *Pleurotus florida* [37,38] have been reported. Literature reports have shown the use of algae *Turbinaria conoides* [39] as reducing agents in AuNPs synthesis. Findings have revealed the synthesis of AuNPs using *Fusarium oxysporum* [40], *Aspergillum sp* [41] and *Trichoderma viride* [42]. Bacteria such as *Klebsiella pneumonia, Salmonella typhi*, *Pseudomonas aeroginosa, Escherichia coli, Rhodopseudomonas capsulate, Streptomyces* sp and *Staphylococcus epidermidis* have been found beneficiary in AuNPs synthesis [43]. List of plant extracts that have been reported for the synthesis of AuNPs are presented in Tables 1 and 2.

**Table 1:** Characterization analysis of some biosynthesized AuNPs using plant sources.

S/N	Plants name	Plants parts	SPR peak (nm)	Functional group prediction		Techniques for Morphological Assessment	Shape	Size	Ref.
1	*Alternanthera bettzickiana*	Leaf	520	3412	O–H	EDX, FTIR, TEM, SEM, UV, XRD	Spherical	60–80	[78]
				2927	C–H				
				1758	C=O				
				1454	C–C				
				1327	N–O				
				1250	C–N				
2	*Musa acuminata colla*	Flower	540	3421	O–H	EDX, UV,XRD,	spherical	10–16	[79]
				2924 2855	C–H				
				2357	C–N				
				1642	C=O				
				1549	C–C				
3	*Flammulina velutipes*	Fruit	563	3357	O–H	FESEM, AFM,UV, DLS, XRD, FTIR	triangular	74.32	[49]
				2950	C–H				
				1634	C–N				
				1389	C=O				
4	*Persea americana*	Oil	520	3009	O–H	TEM, XRD, UV,FTIR,DLS	decahedral	48.8	[80]
				29222853	C–H				
				1743	C=O				
				1653	C=C				
5	*Galaxaura elongata*	-	500–600	3291	O–H	FTIR,TEM,ZP,UV	Spherical, triangular	3.85–77.13	[81]
				2158	C–N				
				1634	C=C				
6	*Mentha arvensis*	-	538	3450	O–H	FESEM,UV,FTIR,DLS	spherical	34	[82]
				1630	C=O				
7	*Pelargonium hirsutum*	-	537	3500	O–H	FESEM,UV,FTIR,DLS	spherical	33.80	[82]
				1624	C=O				
8	*Aegle marmelos*	Fruit	519	3440–3367	O–H	UV,TEM,ZP,FTIR, EDX	Spherical	18	[83]
				2800–3000	C–H				
				1620–1606	N–H				
9	*Eugenia jambolana*	Fruit	523	3440–3367	O–H	UV,TEM,ZP,FTIR, EDX	Spherical	24	[83]
				2800–3000	C–H				
				1620–1606	N–H				
10	*Annona muricata*	Fruit	526	3440–3367	O–H	UV,TEM,ZP,FTIR,SAED,EDX	Spherical	16	[83]
				2800–3000	C–H				
				1620–1606	N–H				
11	*Hygrophila spinosa*	-	540	3263	O–H	UV,EDX,TEM, FTIR, XRD,SEM,ZP	Polygonal, rod	68	[84]
				2926	C–H				
				1716	C=O				
12	*Vitis vinifera*	juice	557	975–3650	O–H	TEM,FTIR,UV	Spherical	82	[85]
				1503–1687	C=O				
13	*Hylocereus undatus*	pulp	560	3451	O–H	TEM,FTIR,UV,XRD	spherical, triangular	0–20	[86]
				2922	C–H				
				1640	C=O				
14	*Gracilaria verrucosa*	Whole plant	520	3448	O–H	TEM,FTIR,UV,XRD,ZP	Spherical	20–80	[87]
				2077	C–H				
				1637	C=O				
15	*Mentha piperita*	Leaf	540	3399	N–H	UV,DLS,SEM,FTIR,FESEM	hexagonal	40–60	[88]
				2135	C–H				
				1645	C=O				
16	*Commiphora wightii*	Leaf	533			EDX,ERD,TEM,UV,FTIR	Triangular, hexagonal	20	[68]
17	*Juglans regia*	husk	550	-	-	XRD,EDX,TEM,FTIR,UV	Spherical	10–30	[89]
18	*Dracaena draco*	Leaf	554–532	3600–3200	O–H	UV,FTIR,TEM,EDX,ZP	Spherical	8–30	[90]
				2900–2800	C–H				
				1725-1720	C=O				
				1612 1640	C=O				
19	*Justicia adhatoda*	Leaf	537	-	-	UV,SEM,EDX	Spherical	13–57	[47]
20	*Sansevieria roxburghiana*	Leaf	560	3415	N–H.O–H	EDX,XRD,UV,FTIR.TEM,DLS,	spherical	25	[91]
				1585	C=C				
				1067	C–N				
21	*Allium cepa*	Peel	535	3631	O–H	UV,FESEM,EDX,XRD, FTIR	spherical triangular	45.42	[92]
				1645	C=O				
				1321	C–N				
22	*Eucommia ulmoides*	Bark	547–536	3776	O–H	EDX,XRD,DLSHRTEM,UV,FTIR	Spherical	18.2	[93]
				3584	N–H				
				3116 2910	C–H				
				1643	C=O				
				1512	C=C				
23	*Muntingia calabura*	Fruit	531	3430	O–H	UV,FTIR,DLS,TEM	spherical and oval	27	[94]
				2962	C=O				
				1617	C=C				
24	*Agave potatorum*	Leaf	540	-	-	UV, HTREM,XRD	pseudospherical	14	[95]
25	*Mariposa Christia Vespertillonis*	Leaf	550	-	-	UV,TEM	Irregular	50–70	[96]
26	*Zostera noltii*	-	550	-	-	TEM,EDX	Spherical	20–35	[97]
27	*Pleurotus cornucopiae*	-	540	3303	O–H	HRTEM, FTIR, UV, EDX	Spherical	16–91	[65]
				2121	C–N				
				1637	C=O				
28	*pueraria lobata*		529	3529–3232	O–H	FTIR, UV,TEM,XRD	Spherical	18	[66]
				1628	C=O

**Table 2:** Applications of AuNPs synthesized from plants extracts.

S/N	Plants name	Plants part	Salt/precursor	Applications	Activities	Ref
1	*Cistus incanus.*	Leaf	HAuCl_4_	luminescence properties	The obtained nanoparticles exhibit good multiphoton-excited luminescence properties.	[111]
2	*Alcea rosea*	Leaf	HAuCl_4_	radical scavenging and catalytic activities	The obtained AuNPs exhibited antioxidant activity against DPPH and ABTS radicals, and catalytic activity in degradation of 4-nitrophenol pollutant	[112]
3	*Punica granatum*	Peel	AuCl_3_	Water Purification	It exhibit suitable activity for water purification.	[98]
4	*Juglans regia*	Bark	HAuCl_4_	Cytotoxicity	It enhance the toxicity towards CTX TNA2 cells than free zonisamide.	[113]
5	*Xanthium strumarium L.*	Fruit	HAuCl_4_	Anti-allergic rhinitis effect	The biosynthesized AuNPs considerably inhibit the allergic inflammation in mice models and could function as anti-allergic rhinitis agents	[114]
6	*Caulerpa racemosa*	Whole plant	HAuCl_4_	Cytoxicity activities	The synthesized AuNPs effectively inhibit the growth of human colon adenocarcinoma (HT-29) cells,	[115]
7	*Vitis vinifera*	Peel	HAuCl_4_	cytotoxicity	the cytotoxicity of the gold nanoparticles against skin cancer A431 cell lines	[104]
8	*Eucalyptus globulus*	bark	HAuCl_4_	Environmental pollution	AuNPs demostrared an eco-friendly reduction conditions	[116]
9	*Citrus limon*	-	HAuCl_4_	Antibacterial Activity	The Au-NPs showed great potential in inhibiting the activities of *Staphylococcus aureus* and *Pseudomonas aeruginosa* by damaging their activity	[117]
10	*Croton caudatus Geisel*	Leaf	HAuCl_4_	Antimicrobial and antioxidant activities	It shows significance Antioxidant and antimicrobial activities.	[118]
11	*Coccinia grandis*	Bark	HAuCl_4_	cytotoxicity	NAC - Au NPs showed no tocity against fibroblast cells	[119]
12	*Halymenia dilatata*	-	HAuCl_4_	antioxidant, anti-cancer and antibacterial activities	Hd-AuNPs exhibit commendable antioxidant, anti-cancer and antibacterial activities	[120]
13	*Capsicum annuum*	Pulp	HAuCl_4_	Catalytic activity.	The GNPs synthesized showed potential catalytic activity.	[121]
14	*Ruellia tuberosa,* *Phyllanthus acidus*	Leaves, wing	HAuCl_4_	enzyme activity	It enhanced the enzyme activity on α-amylase, cellulase, and xylanase	[122]
15	*Cassytha filiformis*	Leaf	HAuCl_3._H_2_O	biodegradation	It shows promising photocatalytic degradation of cationic dye	[123]
16	*Alpinia nigra*	Leaf	HAuCl_3._H_2_O	biodegradation	The synthesized AuNPs in the presence of sunlight catalyzed the degradation of the Methyl Orange and Rhodamine B with percent degradation of 83.25% and 87.64% respectively	[124]

DPPH = 2,2-diphenyl-1-picryl-hydrazyl-hydrate, ABTS = 2,2′-azino-bis(3-ethylbenzothiazoline-6-sulfonic acid), HAuCl_4_ = gold(iii)chloride hydrate, HAuCl_4_ = Chloroauric acid.

**Mechanism for the synthesis of AuNPs using plant extracts**

**Figure 1:**
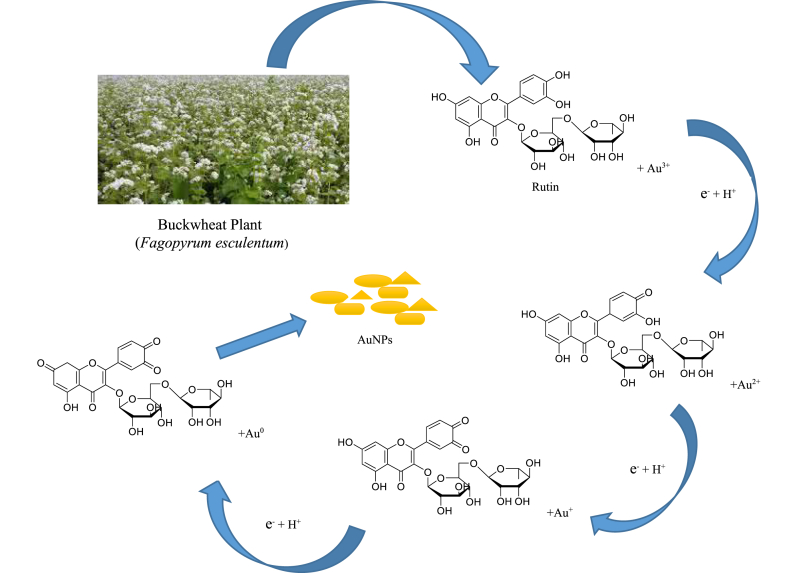
Mechanism of AuNPs synthesis using Plant extracts.

The importance of plant extracts in the synthesis of AuNPs was presented in the reaction mechanism given in Figure 1. The reduction of gold salt to gold nanoparticles (Au^3+^→Au^0^) using plant extract was possible due to the presence of some phytochemicals found in the plant's extract as illustrated with equation I-III. Such phytochemicals have the potential of donating the needed electrons for the relative conversion of (Au^3+^→Au^0^). The presence of hydroxyl functional groups in plant extract enhanced the interaction of –OH with Au^3+^ ions to form gold complexes which can be reduced to a stable Au^0^ [44].

(1) Au^3+^ + e^−^ → Au^2+^

(2) Au^2+^ + e^−^ → Au^+^

(3) Au^+^ + e^−^ → Au^0+^

## 3 Characterization of nanoparticles

The main parameters studied when AuNPs are characterized are surface charge, crystallinity, morphology and particle size. The various techniques used for the evaluation and quantification of these parameters are summarized in detail below.

### 3.1 Nanoparticle formation analysis

UV-visible spectroscopic analysis is used to ascertain the formation of AuNPs by measuring the surface plasmon resonance [SPR] and assessing the collective oscillations of conduction bands of electrons generated by electromagnetic waves [45]. The use of UV-visible spectroscopic analysis have aided the determination of some morphological features such as stability, size, structure and shape of AuNPs [46]. Each metal nanoparticles has a precise absorbance band in representative spectra when incident light encounter some resonance with the conduction band electrons on the surface of the nanoparticles. Due to the excitation mode of the surface plasmons, AuNPs have an absorbance peak in the range of 500–550 nm [47]. The shift in the SPR peak towards the blue shift (lower frequency) had been reported to indicate decrease in size of AuNPs size whereas the red shift (lower frequency) signifies an increase in AuNPs size. The red shift occurs when the crystal size grows after nucleation and the distance between valence band increases [48]. Comprehensive information on the use of UV-visible spectroscopy in the confirmation of AuNPs formation is described in Table 1.

### 3.2 Functional group determination

Fourier-transformed infrared spectroscopy (FTIR) is used for the determination of the functional groups that induced the reduction of metal precursors [49]. To achieve this, the technique measures the absorption of electromagnetic radiation in the infrared region of wavelengths (4000-600 cm-1). FTIR spectrum describes the location of bands in relation to the nature of bonds and definite functional groups [49]. The peaks at the following wavelength specify functional groups associated with AuNPs synthesis. 3400-3380 for Ν-Η amines, 3570-3200 for O–H of alcohols, 3130-3070 cm^−1^ for aromatic C–H, 2970-2860 cm^−1^ for Methyl C–H, 2300-2150 cm^−1^ for C–N of nitriles, 1790-1690 for carbonyl –C=O, 1680-1620 cm^−1^ for C=C alkenes and 1470-1370 cm^−1^ for Methyl C–H as showed in Table 1. Phytochemicals with the listed functional groups are expected to be capable of reducing gold precursor because they are capable of donating electrons needed for the reduction of gold ions to a lower oxidation state [50]. Comprehensive information from previous literatures on the use of FTIR as characterization technique in green synthesis of AuNPs are presented in Table 1.

### 3.3 Morphology and particle size determination

The effective applications of AuNPs depend largely on their morphological properties and particle size distribution. Hence the need for the evaluation of these feacture via characterization are very significant. The following microscopy techniques; transmission electron microscopy (TEM), scanning electron microscope (SEM) and Atomic Force Microscopy (AFM) are mostly used to measure the aforementioned properties [51].

#### 3.3.1 Transmission electron microscopy (TEM/HRTEM)

TEM is a commonly used techniques for the determination of shape, morphology and size of AuNPs. This technique is based on the interaction between a thin sample and a uniform current density electron beam (energies in the range of 150 to 60 keV). When the electron beam come in contact with the sample, most of the electrons are transmitted, while the rest are scattered. Data generated from the transmitted electrons are used for the production of the TEM image [52]. The degree of such interaction depends on size, elemental composition and sample density. TEM is rampantly used for size and shape analysis. TEM does not only offers direct images of nanoparticles it equally gives an accurate estimation of their homogeneity [52,53]. Nevertheless, common limitations of TEM technique are; it damage samples that cannot withstand the vacuum pressure of the microscope, it produce misleading images as a result of orientation effects, sample preparation is difficult, it waste time because the samples must be ultrathin for electron transmittance and it cannot be used for the quantification of large number of particles [52,54]. High-resolution transmission electron microscopy (HRTEM) is another version of TEM which allows the combination of both the transmitted and scattered electrons for the production of its image because it make use of phase-contrast imaging [55]. This technique offer more benefits when compared with TEM it generating high resolution images and can also characterize the internal structure of nanoparticles [56]. Detailed findings on the use of TEM and HRTEM in the determination of size, shape and morphology of AuNPs is presented in Table 1.

#### 3.3.2 Scanning electron microscopy (SEM)

SEM is another microscopic technique that enhances the morphological characterization of nanoparticles through direct visualization [57]. When AuNPs are exposed to electron beams they generate signals that are recorded by the detector which gives information about the chemical composition, external morphology, orientation and crystalline structure of AuNPs [58]. It exhibit several benefits such as prevention of sample destruction, it permit visualization of morphological properties in liquid state which prevent destruction of polymeric nanoparticles because complete drying could alter their morphological identities. Despite these advantages the following are its demerits; it doesn't offer accurate information about the true population average and size distribution, it is time wasting, costly to operate and it damage samples that cannot withstand vacuum pressure [52]. The applications of this technique in determination of the size and shape of AuNPs are listed in Table 1.

#### 3.3.3 Atomic force microscopy

Atomic Force Microscopy (AFM) are used for identification of the morphological identities of nanoparticles. The difference between this technique and SEM/TEM is the production of three-dimensional images which enable the evaluation of the particle volume and height [59]. This technique is based on the physical scanning of nanoparticles at submicron level via a probe tip [60]. With the aid of AFM, the size (length, width, and height), surface texture and morphology of nanoparticles can be determined with software based image processing [61]. This technique has ability to produce images of nanoparticles without any further treatment [62]. Reports on the morphological characterization of AuNPs via AFM are presented in Table 1.

### 3.4 Dynamic light scattering

Dynamic Light Scattering (DLS) is a popular technique used for estimating particle size distribution. DLS has been reported to be a good technique for the measurement of Brownian nanoparticles sizes in colloidal suspensions [63]. DLS make use of diffusion coefficient in the computation of size distribution and nanoparticles motion [64]. Reports on the application of DLS in size distribution of AuNPs are stated in Table 1.

### 3.5 Elemental composition

Energy Dispersive X-Ray Spectra (EDX) had been reported by several researchers to be a good technique for the estimation of elemental compositions and purity determination in AuNPs synthesis [65]. This is achieved from the quantification of X-rays emissions from nanoparticles after they are bombarded by electron beam [46]. EDX detector are connected to SEM for the detection of number of X-rays emitted so as to balance the difference in energy of two electrons. The energy generated by the emitted X-ray is a characteristic property of the element, which when qualitatively and quantitatively analyzed gives the composition of the element present in the synthesized AuNPs [66].

### 3.6 Crystallinity analysis

The crystallinity of nanoparticles is determine via X-ray Diffraction (XRD) technique [67]. This technique measures the diffraction angle obtained from the structure and lattice parameters of the diffracted powder samples when X-ray beam incident on them. The particle sizes are then estimated according to the width of the X-ray peaks via the Scherrer formula in equation iv [68].

(4) D = kλ / β cos2θ

where D represent the average thickness of crystalline grains at the crystal plane (n), K denote the Scherrer constant (0.89), β stand for the Full-Width Half Maximum, θ represent diffraction angle and λ signify X-ray wavelength (0.15418 nm).

### 3.7 Raman spectroscopy/surface-enhanced Raman spectroscopy (SERS)

SERS technique incorporates nanotechnology into normal Raman spectroscopy. The normal Raman technique depends on the inelastic scattering irradiated analyte produced by laser. Therefore, any polarizable sample under laser irradiation will be detected by Raman spectroscopy [69]. SERS was design to solve some problems associated with Raman spectroscopy such as difficulty in determination of samples at low concentrations and poor fluorescence interference. SERS has been reported to display a remarkable advantages of rapid identification of NPs from their dissolved ions and bulk counterparts over other techniques [69]. SERS has been successfully used for the size determination of some nanoparticles [70]. The use of SERS imaging in intracellular and extracellular mapping of AuNPs synthesized using green algae *Pseudokirchneriella subcapitata* has been documented [71] Lahr et al. reported that SERS is useful in tracking AuNPs fate in microfluidic paper-based analytical devices [72]. AuNPs toxicity against microorganisms had been estimated with the use of SERS [73]. The adsorption of molecules onto AuNPs surfaces had been monitored via SERS [74].

### 3.8 Zeta potential

Zeta Potential (ZP) is a prominent analytical tool used for the determination of surface charge of AuNPs in colloidal solution. The surface of a charged nanoparticles attracts a tiny layer of oppositely charge ions and binds firmly to form a thin liquid layer. The diffusion of the particles through the solution involve an outer diffuse layer which consists of roughly associated ions thereby forming an electrical double layer [75]. ZP values are used in predicting the stability of nanoparticles and the values ranges from +100 to -100 mV. AuNPs with ZP values greater than +25 mV or lesser than –25 mV are regarded as been stable. Lower ZP values are associated with aggregation or coagulation as a result of van der Waals attraction [76]. The ZP values in the range of -33.59 to -29.1 mV has been reported for AuNPs synthesized using *Leucosidea sericea* [77].

## 4 Applications of AuNPs synthesized from plant extracts

### 4.1 Catalytic application

The aquatic ecosystem are polluted when municipal and industrial effluent are discharged into them, this happening had cause enormous environmental and health menaces. Several techniques such as osmotic pressure, adsorption and coagulation have been utilized in treating the effects of wastes and effluents from the aquatic ecosystem but each techniques have some unique merits and demerits. Interestingly, the photocatalytic treatment using biosynthesized AuNPs from plant sources provides a cost effective and environmental friendly approach of solving this problem [98]. The effective use of AuNPs as photocatalytic agent in degradation of organic pollutants are linked to their large surface area or the quantum confinement effects [99]. The catalytic efficiency of AuNPs biosynthesized from *Mimosa tenuiflora* extract has been stated [100]. Also the investigation of biosynthesized AuNPs using *Trichoderma sp* as catalytic agent in degradation of azo dyes has been established [101]. Furthermore, AuNPs Phyto-synthesized from *Delonix regia* leaf extract demonstrated potential catalytic activity [102]. More relevant information on the catalytic application of AuNPs mediated from plant extracts are presented in Table 2.

**Possible catalytic mechanism**

**Figure 2:**
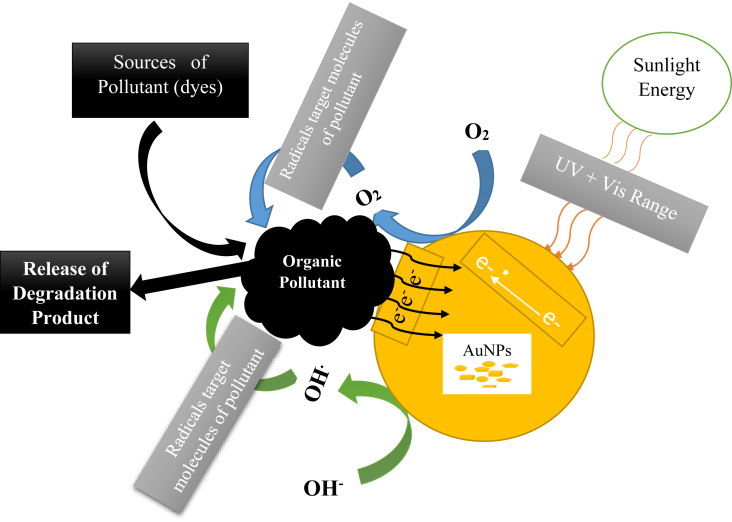
Schematic representation of degradation of some dyes using the AuNPs.

Two mechanisms have been proposed for the photocatalytic efficiency of AuNPs; Firstly, AuNPs are capable of inducing localized surface plasmon resonance (LSPR) by absorption of visible light with equivalent wavelengths of their plasmonic absorption bands. The electromagnetic field associated with LSPR can enhance the production and separation of electron/hole pairs in the semiconductor. Secondly, AuNPs can serve as electron sinks to attract photogenerated electrons, thereby causing an effectual separation of electron/hole pairs. However, the aforementioned mechanisms depend on the AuNPs sizes, larger-sized AuNPs follows the LSPR effects whereas smaller-sized AuNPs generally behaves as electron sinks [28]. The schematic representation of the degradation mechanism of AuNPs is showed in Figure 2.

### 4.2 Anticancer application

**Figure 3:**
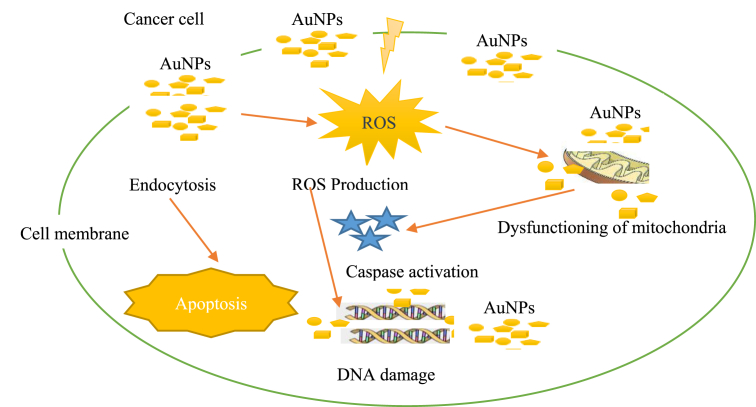
Schematic representation of the anticancer activity of AuNPs.

Biosynthesized AuNPs are found to possess distinctive anticancer activities, because biomolecules can easily absorb to their surfaces making it superior to other materials in inhibiting the growth of cancer cell [103]. AuNPs produced using plants were reported to have huge anticancer potential since they demonstrated toxic activity against cancer cells [104]. The efficacy of AuNPs produced using *Vitis vinifera* peel showed commendable biocompatibility towards normal human cell line and selective toxicity against skin cancer A431 cell lines rendering it a good anticancer agent [105]. The cytotoxic investigation of AuNPs against the growth of human breast cancer cells (MCF-7) showed 60% cell death at concentrations of 120 mg/ml. It was also observed that the cytotoxicity increased with the increase in concentration of AuNPs [83]. Findings had shown that the anticancer efficiency of AuNPs depends on the stabilizing agent and size [106]. Other useful information on the anticancer application of green synthesized AuNPs are showed in Table 2. The Schematic representation of the anticancer activity of AuNPs is showed in Figure 3.

### 4.3 Antioxidant application

**Figure 4:**
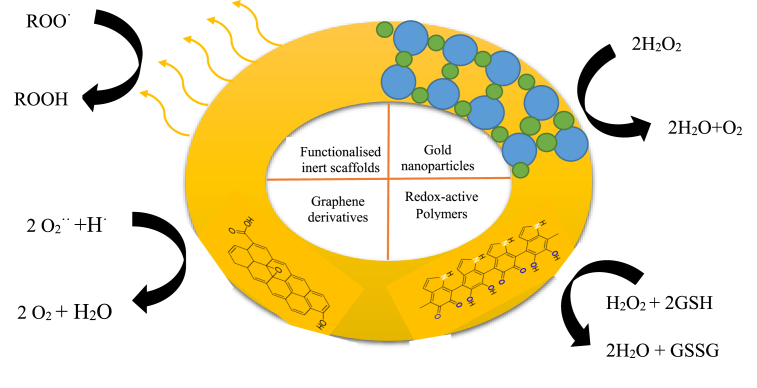
Schematic representation of the antioxidant activity of AuNPs.

The results obtained from 2,2-diphenyl-1-picryl-hydrazyl-hydrate (DPPH) assay of AuNPs mediated from *Alium cepa* displayed moderate antioxidant activity of 14.44% at 100 μg/ml while the reference standard showed scavenging activity of 36.54% at same concentrations when compared [92]. The DPPH scavenging potential of phytosynthesized AuNPs are linked to the presence of secondary metabolites in plant extracts [107]. The antioxidant analysis of AuNPs prepared from avocado oil showed an antioxidant efficiency of 30.49 % at 40 μL. The antioxidant efficiency was ascribed to the functionalization of AuNPs with some organic constituents derived from the avocado oil [80]. Reports from previous studies have claimed that the interaction of AuNPs with free radicals are influenced by some factors such as specific surface, concentration and size. Furthermore, the authors equally stated that larger size AuNPs exhibits lower antioxidant potency when compared with smaller size of AuNPs [80,84]. Relevant information on the antioxidant efficiency of AuNPs synthesized using plant extracts are presented in Table 2. The Schematic representation of the antioxidant activity of AuNPs is showed in Figure 4.

### 4.4 Antimicrobial application

**Figure 5:**
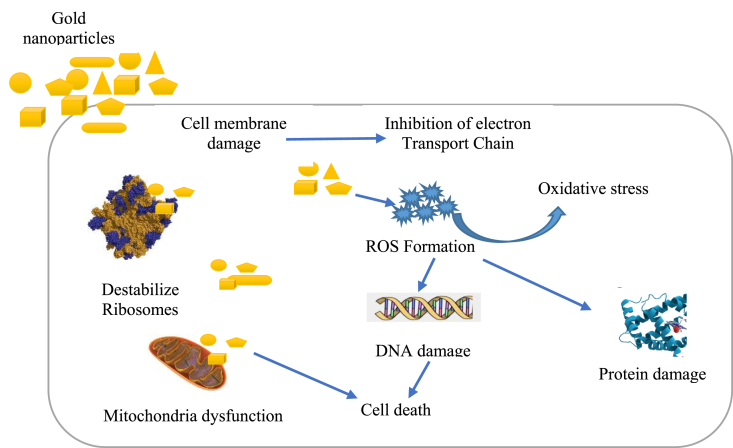
Schematic representation of the antibacterial mechanism of AuNPs.

AuNPs synthesized from the powder of *Galaxaura elongata* was found to be an effective antibacterial agents judging from the inhibition zones of 13.5 and 13 mm demonstrated against the growth of *E. coli* and *K. pneumoniae* respectively [81]. The proteomic study of AuNPs against the growth of *E. coli* cells showed an alterations in the expression of heat sock protein even at short exposure [108]. Green synthesis of AuNPs using *Alternanthera bettzickiana* leaf extract demonstrated a significant antibacterial effect rendering it a promising antibacterial agent. Interestingly, report had shown that biosynthesized AuNPs displayed more efficient antibacterial activity than chemically synthesized AuNPs when compared, such antibacterial potency may be due to the synergistic effect of the plant extracts [78]. Several finding have shown that AuNPs synthesized from plant extracts exhibit remarkable antimicrobial activities against several microbes [109,110]. Additional findings on the antimicrobial application of phytosynthesized AuNPs are showed in Table 2. The Schematic representation of the antibacterial mechanism of AuNPs is showed in Figure 5.

## 5 The challenges and future of AuNPs for bioremediation and biomedical applications

The applications of AuNPs is on a widespread across the globe as its usefulness in most life fields such as dye, electronics, textile, food, medicine and other industries are increasing on daily basis [125]. Recently nanoscience and nanotechnology are providing remarkable and promising bioremediation and biomedical applications than what is recorded in previous decades. The quest to search for a lasting solution to improve human health status through disease diagnosis and production of novel medicine had made Nano research a very relevant field of study [126]. This move will continue to grow rapidly with provision of prominent contributions to public health and other fields [127]. However, the safety level of AuNPs in bioremediation and biomedical applications are becoming worrisome and its raising some future challenges due to nanotoxicity because the interaction of AuNPs with biological systems are govern by several factors such as cell type, uptake routes or directing different organelles which might sometimes be harmful [128]. Also investigation of the mechanism of interaction, degradation and bioaccumulation of metal nanoparticles in living organism had revealed that the physico-chemical properties (particle sizes and shapes) of nanoparticles influences their toxicological behaviors greatly [129]. Surface modification and dosage regulation of nanoparticles had been documented as appropriate route to enhance nanoparticles efficiency, safety and could also cause the reduction of their negative impacts [130]. Scaling-up the synthesis of AuNPs from laboratory approach to meet the commercial demand is also a great challenge to bioremediation and biomedical researchers because particles size control and modification are easier at the laboratory scale due to nanoparticles composition when compared with industrial scale [131]. A comprehensive understanding of the reaction mechanisms, intracellular mechanisms, dosage, route trafficking and biological activity of AuNPs are highly required to proceed in this facet.

## 6 Conclusion

Considering the various methods of synthesizing AuNPs, the phytosynthesis based method is regarded as the best, due to its invincible benefits in terms of cost effectiveness, possibility of been scale up for large scale production, reproducibility, environmental-friendly, immunological behavior, easy operation, tunability and relative abundance of plants. In this review, the green synthesis and various techniques used for characterization of phytosynthesized AuNPs were properly described in detail. The tremendous potential of phytosynthsized AuNPs in bioremediation and biomedical applications were comprehensively highlighted. These remarkable potentials are attributed to the functional groups found in plant extracts and morphological identities of AuNPs. Future studies on green synthesis of AuNPs should focus on providing more relevant information on factors influencing its synthesis, durable mode of preservation of extracts for longer period, more sophisticated and easy to operate techniques for characterization of AuNPs so as to enhance the wide applications of phytosynthesized AuNPs in more field of sciences and technologies.

## Declarations

### Author contribution statement

All authors listed have significantly contributed to the development and the writing of this article.

### Funding statement

This research did not receive any specific grant from funding agencies in the public, commercial, or not-for-profit sectors.

### Data availability statement

Data will be made available on request.

### Declaration of interests statement

The authors declare no conflict of interest.

### Additional information

No additional information is available for this paper.
